# Publicly Available Datasets for Artificial Intelligence in Neurosurgery: A Systematic Review

**DOI:** 10.3390/jcm14165674

**Published:** 2025-08-11

**Authors:** Bianca Chan, Brandon Kim, Ethan Schonfeld, George Nageeb, Aaradhya Pant, Adam Sjoholm, Ravi Medikonda, Ummey Hani, Anand Veeravagu

**Affiliations:** 1 Stanford Neurosurgical Artificial Intelligence and Machine Learning Laboratory, Stanford School of Medicine, Stanford University, Stanford, CA 94305, USA; cbianca@stanford.edu (B.C.); bkim7@stanford.edu (B.K.); gnageeb@stanford.edu (G.N.); rdpant@stanford.edu (A.P.); macsjoh@stanford.edu (A.S.); uhani@stanford.edu (U.H.); anand.veeravagu@stanford.edu (A.V.); 2 Stanford University School of Medicine, Stanford University, Stanford, CA 94305, USA; 3 Department of Neurosurgery, Stanford University School of Medicine, Stanford University, Stanford, CA 94305, USA; rmediko1@stanford.edu

**Keywords:** neurosurgery, machine learning, dataset, spine, surgery, validation, outcome, metrics

## Abstract

**Introduction**: The advancement of artificial intelligence (AI) in neurosurgery is dependent on high quality, large, labeled datasets. Labeled neurosurgical datasets are rare, driven by the high expertise required for labeling neurosurgical data. A comprehensive resource overviewing available datasets for AI in neurosurgery is essential to identify areas for potential model building and areas of needed data construction. **Methods**: We conducted a systematic review according to PRISMA guidelines to identify publicly available neurosurgical datasets suitable for machine learning. A PubMed search on 8 February 2025, yielded 267 articles, of which 86 met inclusion criteria. Each study was reviewed to extract dataset characteristics, model development details, validation status, availability, and citation impact. **Results**: Among the 86 included studies, 83.7% focused on spine pathology, with tumor (3.5%), vascular (4.7%), and trauma (7.0%) comprising the remaining. The majority of datasets were image-based, particularly X-ray (37.2%), MRI (29.1%), and CT (20.9%). Label types included segmentation (36.0%), diagnosis (26.7%), and detection/localization (20.9%), with only 2.3% including outcome labels. While 97.7% of studies reported training a model, only 22.6% performed external validation, 20.2% shared code, and just 7.1% provided public applications. Accuracy was the most frequently reported performance metric, even for segmentation tasks, where only 60% of studies used the Dice score metric. Studies often lacked task-appropriate evaluation metrics. **Conclusions**: We conducted a systematic review to capture all publicly accessible datasets that can be applied to build AI models for neurosurgery. Current datasets are heavily skewed towards spine imaging and lack both clinical patient specific and outcomes information. Provided baseline models from these datasets are limited by poor external validation, lack of reproducibility, and reliance on suboptimal evaluation metrics. Future efforts should prioritize developing multi-institutional datasets with outcome labels, validated models, public access, and domain diversity to accelerate the safe and effective integration of AI into neurosurgical care.

## 1. Introduction

The rise of deep learning and large language models (LLMs) has recently drastically expanded artificial intelligence (AI) translation to various applications of biomedical science and healthcare, including drug discovery, diagnosis, treatment planning, intervention, education, and administrative processes. Already having transformed many industries, the potential for AI translation to medicine, and specifically neurosurgery, is enormous. AI has already been developed in neurosurgery to augment decision making and precision during preoperative, intraoperative, and postoperative stages. For example, models trained using a large database of operative parameters, patient imaging, and post-operative outcomes, pre-operatively suggest which pedicle screw parameters are optimal for a specific patient. This technology was found to improve the rate of the post-operative acceptable spinopelvic alignment parameter in complex deformity patients, demonstrating the power of decision making support tools [1].

The largest hurdle for developing these tools for neurosurgery is that of having a sufficient amount of labeled data to train the models. Deep learning and specifically LLMs and foundation models require large datasets even to finetune off the shelf pre-trained models [2]. Neurosurgery is a data rich environment; consider, for example, the enormous data stored on recorded microscopy videos or unstructured data in patient charts. The limitation is that neurosurgical data is extremely difficult to label. Traditional dataset development in other domains commonly gather a large data amount and then use outside labelers to generate the ground truth labels that can be used for training models on specific tasks. The external data labeling market in 2020 was expected to triple in size by 2024, reflecting the increased need and utilization for labeled data [3,4]. In neurosurgery, clinical expertise is required to generate labels and, therefore, external label generation would be hugely expensive and non-practical. Therefore, high quality datasets for translational ML model development are a limiting factor in AI for neurosurgery.

With the growing clinical use and research development of AI in neurosurgery, a comprehensive resource detailing available datasets in neurosurgery is needed. Such a resource would both function as a resource for researchers seeking to build AI tools for neurosurgery as well as function to identify which areas of neurosurgery most require intentional construction of appropriate datasets to allow future AI development. To build this resource, we conducted a systematic review to capture all publicly accessible datasets that can be applied to build AI models for neurosurgery. We further summarize trends in dataset development in neurosurgery, focusing on which factors allow for most impact to guide the development of robust future datasets that stand to catalyze AI translation to neurosurgery and improved patient care.

## 2. Methods

### 2.1. Search Strategy

This systematic review was conducted in alignment with the Preferred Reporting Items for Systematic Reviews and Meta-Analyses (PRISMA) guidelines. A comprehensive literature search was initially performed in the PubMed database via pubmed.ncbi.nlm.nih.gov on 8 February 2025. The following exact query was applied: *data release [Title/Abstract] OR “novel data” [Title/Abstract] OR “primary dataset” [Title/Abstract] OR “dataset” [Title/Abstract]) AND (“machine learning” [Title/Abstract] AND “artificial intelligence” [Title/Abstract] OR “AI” [Title/Abstract] OR “deep learning” [Title/Abstract] OR “ML” [Title/Abstract] OR “Neural Networks” [Title/Abstract]) AND (“neurosurgery” [MeSH Terms] OR “neurosurgical” [Title/Abstract] OR “vascular neurosurgery” [Title/Abstract] OR “neurooncology” [Title/Abstract] OR “functional neurosurgery” [Title/Abstract] OR “spine” [Title/Abstract] OR “TBI” [Title/Abstract] OR “neurosurgical care” [Title/Abstract]) AND (“2016/01/01” [Date—Publication]: “3000” [Date—Publication])).*

### 2.2. Screening

**Figure 1:**
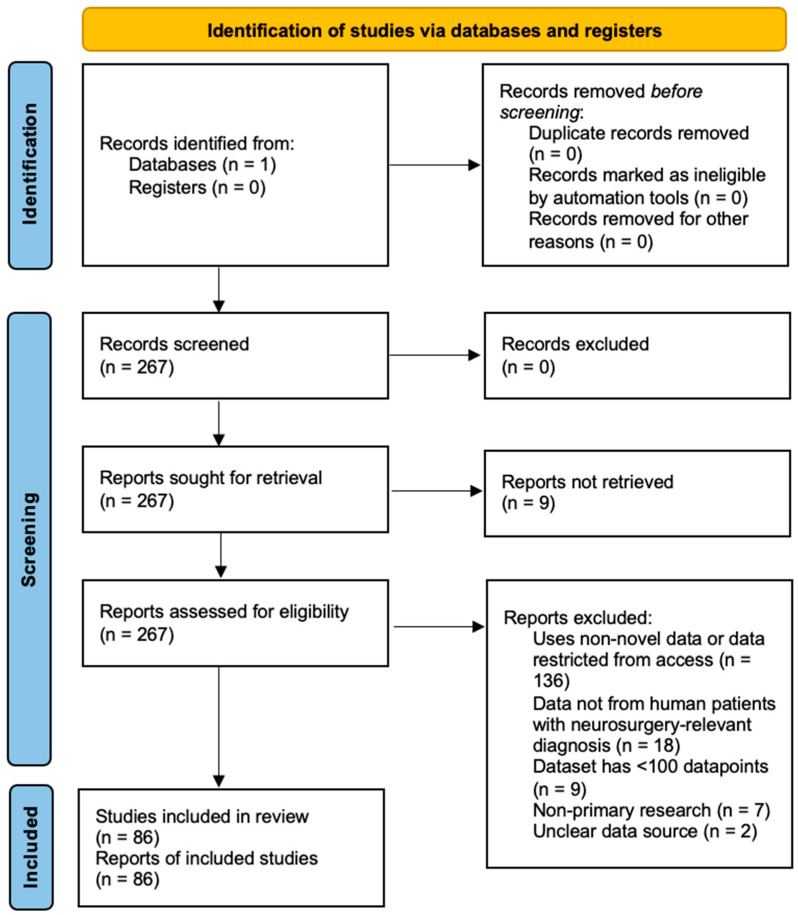
PRISMA diagram of article screening and selection.

All resulting studies from the search strategy were manually and individually reviewed by two researchers (B.C. and B.K.) in parallel. The following inclusion criteria was used: (1) publication from 01/01/2016 and onward, (2) release of novel data, not a secondary analysis or analysis of previously released data, (3) already publicly available data or can apply for access, (4) data derived from human patients with neurosurgical relevant diagnosis in pre, peri, post-operative care or laboratory samples. The following exclusion criteria was also used: (1) non-English articles, (2) non-primary research, (3) less than 100 data items, (4) non-peer reviewed articles and abstracts. Studies that included <100 patients but 100 or more data items was not excluded due to the increase in few shot learning techniques available. The 100 data item requirement aimed to exclude case series that focus on dedicated projects of dataset development. Furthermore, novel data must comprise the majority (>=50%) of all data used for training for studies that use both private and public datasets. If the split is unclear, the study was excluded. This criteria was included to limit any double-counting of previously published data in our analysis. If data was cited in a previously published study, the article was excluded. If there is no data availability statement for a private dataset, it was assumed that access can be requested from the authors of the article and thus the article is included. This assumption was included so as to avoid missing subdomains of data that the convention may be to request data directly from authors in the subdomain. After articles were independently selected by the screeners based on the above criteria and assumptions, any disagreements between the two screeners were resolved through discussion (Figure 1).

### 2.3. Data Extraction and Analysis

All the data objects from the selected articles were independently extracted by the two authors in parallel (Supplemental Table S1). Basic identifying information obtained included title, publication year, and PubMed ID. To study generalizability and validation of the data/models, we then extracted the following variables: whether the data is labeled, whether the data source is multi-institutional, number of institutions, whether there are institutions that are non-tertiary academic centers, whether a trained model is included, whether the model is externally validated, and whether the model is publicly available (e.g., if code and weights are provided) and has a public application (e.g., available web application). Specifically, to meet the criteria for external validation, the model must be evaluated with data from more than one institution. To study model functionality and impact, we also collected the primary data type, the sample size of the primary data, the label type, the inference class if the paper included a baseline model, performance metrics of the model, and the number of article citations. Any disagreements between the two authors on the information from object extraction were resolved through discussion.

Prior to performing analysis, we classified the primary data type into seven categories (i.e., X-ray, CT, MRI, PET, clinical, sensor, video). We also categorized the label type into seven categories (i.e., diagnosis, segmentation, detection/localization, intervention, grading, image, outcome). Additionally, across all studies, the inference class of the baseline model was classified into eight categories (i.e., diagnosis, detection/localization, segmentation, intervention, measurement, grading, outcome, generative). All baseline models were classified into linear, convolutional neural network (CNN), segmentation, non-transformer NLP, transformer, and generative adversarial network (GAN).

### 2.4. Quality Assessment

To assess the methodological quality and risk of bias of included studies, we developed a custom checklist reflecting critical aspects of dataset and model robustness. Each study was evaluated for inclusion of multi-institutional data, use of external validation, public availability of code, presence of a public-facing application, use of task-appropriate performance metrics, and inclusion of outcome labels. A score from 0 to 6 was assigned based on the number of criteria met. This simple quality score enables readers to gauge the risk of bias and generalizability of each dataset.

## 3. Results

### 3.1. Study Selection

Implementing the described search strategy, we identified 267 articles that pertained to the use of AI or machine learning in the neurosurgical process. After applying our inclusion/exclusion criteria to these articles, a total of 86 studies were included in the final analysis (Figure 1). 181 studies were excluded: 9 full-text articles could not be accessed, 9 had sample sizes under 100, 7 had non-primary research designs, 136 used non-novel or restricted datasets, 2 had unclear data sources, and 18 did not involve neurosurgically relevant human subjects. Each included study was reviewed to extract relevant data. Each variable extracted was recorded and then categorized for analysis.

### 3.2. Study Characteristics

#### 3.2.1. Analysis of Dataset Characteristics

The vast majority of included studies were spine (83.7%) with trauma comprising the second most common category of dataset (7.0%). Image-based datasets were heavily favored among the 86 included studies, with X-ray (37.2%), MRI (29.1%), and CT (20.9%) representing the majority of data types (Table 1). Non-imaging sources such as clinical data (16.3%), video (2.3%), PET (2.3%), and sensor data (1.2%) were noticeably less substantial. Similarly, the most common label types were segmentation (36.0%), diagnosis (26.7%), detection/localization (20.9%), and grading (20.9%), while the most common inference class were diagnosis (42.9%), detection/localization (41.7%), and segmentation (34.5%). Both are consistent with the prevalence of image-based tasks.

The average number of patients per study was 756.4, but this distribution was highly skewed as the SD was 925.8 and median was 100.

Nearly all studies (97.7%) included a trained baseline ML model. Of the included ML models, CNNs was the most common architecture (61.9%), followed by segmentation models (31.0%) and linear models (11.9%). Transformers (3.6%), non-transformer NLP (3.6%), and GANs (1.2%) were less commonly used. Citation counts varied widely, with a median of 6.5 citations and range from 0 to 127.

#### 3.2.2. Analysis of Inference Type by Data Modality

**Figure 2:**
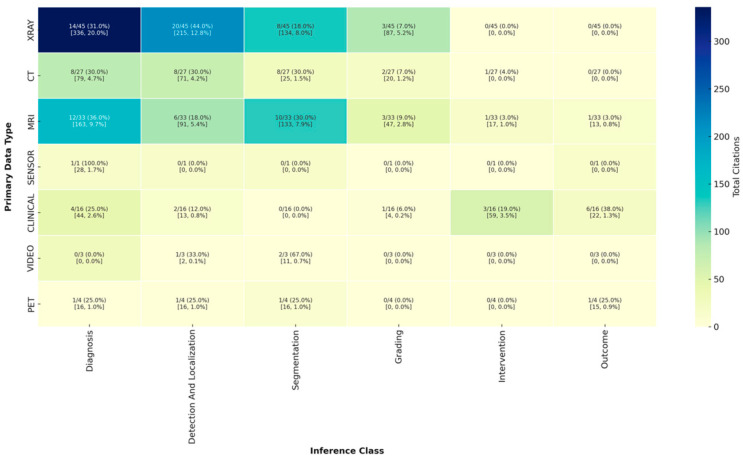
Heatmap of citation counts by primary data type and inference class of baseline provided model.

Diagnosis and detection/localization tasks (particularly with X-ray) accounted for the largest share of citations overall (Figure 2). MRI-based datasets addressing diagnosis and segmentation were also highly cited (163 and 133 citations, respectively), despite representing a smaller fraction of total datasets, underscoring their impact in neurosurgical imaging tasks.

CT datasets showed relatively balanced distribution across diagnosis, detection/localization, and segmentation (each 30.0%), yet had markedly lower total citations (e.g., only 71 citations for detection/localization and 25 for segmentation). Clinical data, while less represented (16 datasets), were uniquely skewed toward outcome prediction (38.0%) and intervention planning (19.0%), and were responsible for a notable 59 citations in intervention and 22 in outcome prediction.

Other modalities such as PET, video, and sensor data were minimally used and had relatively low citation counts. For example, video datasets were focused mostly on segmentation (67.0%) but yielded only 11 total citations. Overall, the most cited inference classes were diagnosis (particularly from X-ray), segmentation (largely from MRI and X-ray), and detection/localization, while grading, intervention, and outcome prediction were underrepresented and cited less frequently—suggesting both a gap in focus and potential areas for growth in high-impact dataset development.

#### 3.2.3. Analysis of Model Validation and Public Application

The overwhelming majority of studies demonstrated minimal or no external validation. This was observed regardless of model type or data type (Table 2 and Table 3). Despite widespread model development, only 22.6% of models were externally validated, just 20.2% made their code publicly available, and just 7.1% developed a functioning public application (Table 1). This indicates a lack of reproducibility and translational ability of these studies. Only 26.7% of studies used multi-institutional data, further indicating the lack of robustness and hurdle for translation. Among architectures, both CNNs and segmentation models were externally validated in 23.1% of studies (Table 2 and Table 3) and made their model public 21.2% and 19.2%, respectively. Their rate for developing public application dropped to 7.7% each, representing a major limitation for clinical translation that even the most robust and validated models were only accessible to the technical and non-clinical audience. Transformer and Non-transformer NLP models showed higher rates but were only based on three studies each. Other model types such as GANs and linear models showed no instances of external testing.

#### 3.2.4. Analysis of Performance Metrics

The use of performance metrics varied widely across different types of inference tasks. Accuracy was the most commonly reported metric overall. It was used in 71% of diagnosis studies, all outcome prediction studies, and nearly half of detection/localization studies (Table 4). Sensitivity and specificity were also frequently reported, especially in diagnostic studies. Sensitivity was reported in 87.1% of diagnosis studies, and specificity was reported in 58.1%. Other metrics such as Gwet’s kappa, IOU, mean absolute error (MAE), and negative predictive value (NPV) were rarely used. Importantly, AUC was only reported in 29% of diagnostic studies, 50% of intervention studies, and 40% of outcome studies, all lower than the use of accuracy for these studies. The use of DICE score in only 60% of segmentation studies, and use of accuracy in 48% of segmentation studies suggests the use of non-task appropriate metrics despite frequent citation (Table 5).

### 3.3. Risk of Bias in Included Studies

Given the focus on dataset characteristics rather than clinical outcomes, a formal risk-of-bias tool (e.g., ROB 2 or ROBINS-I) was not applicable. Instead, study quality was appraised based on dataset novelty, sample size, data source transparency, and use of external validation. Several potential sources of bias were identified based on these criteria (Supplemental Table S2).

### 3.4. Reporting Completeness

Among the 86 studies, 15 (17.4%) lacked critical metadata. Eleven studies did not report the number of patients, and nine studies did not specify whether data were sourced from multiple institutions. These gaps limit dataset reusability and underscore the importance of standard reporting checklists.

### 3.5. Key Findings from High-Impact Studies

In the study, “Deep learning of lumbar spine X-ray for osteopenia and osteoporosis screening: A multicenter retrospective cohort study” with 127 citations (PMID: 32730939, Supplemental Table S1), the authors trained a CNN to classify bone mineral density status using lumbar spine X-ray images [5]. The model demonstrated the ability to distinguish between normal bone density, osteopenia, and osteoporosis utilizing only the image data. Its performance was primarily evaluated using AUC and sensitivity as key metrics. The model’s performance reached AUC of 0.810 to diagnose osteopenia with sensitivity of 85.3%. Their developed model was externally validated across two independent datasets and is publicly available but does not have a public application.

The study, “Can a Deep-learning Model for the Automated Detection of Vertebral Fractures Approach the Performance Level of Human Subspecialists?” with 74 citations (PMID: 33651768, Supplemental Table S1), developed an ensemble of CNNs to automatically detect vertebral fractures on lateral spine radiographs [6]. The model demonstrated excellent ability to identify fractures across thoracic and lumbar regions with similar ability to expert radiologists. Its performance was primarily evaluated using key performance metrics of accuracy, sensitivity, and specificity. The model demonstrated detection of vertebral fractures of the lumbar spine with 93% accuracy, 91% sensitivity, and 93% sensitivity. Additionally, it had stronger performance with distinguishing nonosteoporotic lumbar vertebrae as opposed to normal osteoporotic lumbar vertebrae. The model also had a better detection of lumbar vertebral fractures as opposed to thoracic vertebral fractures. The model was externally validated and was made into a publicly available application which can be utilized as a tool to support radiologists.

The study, “Feasibility of Deep Learning Algorithms for Reporting in Routine Spine Magnetic Resonance Imaging” with 44 citations (PMID: 33298549, Supplemental Table S1), developed a CNN to detect and report common lumbar pathologies and injuries on MRI scans [7]. Specifically, their model showed promise to identify foraminal stenosis, central canal stenosis, and disc herniation. Its performance was assessed primarily using accuracy, sensitivity, and specificity as key metrics. The model reported detection of foraminal stenosis with an accuracy of 81%, sensitivity of 72.4%, and specificity of 83.1%, detection of central stenosis with an accuracy of 86.2%, sensitivity of 91.1%, and specificity of 82.5%, and detection of disc herniation with an accuracy of 85.2%, sensitivity of 81.8%, and specificity of 87.4%. Their findings were internally validated and support the feasibility of using deep learning algorithms for routine MRI reporting.

## 4. Discussion

The availability of large, labeled datasets of clinical, imaging, and outcome patient data is cornerstone to the translation of AI models for neurosurgery. The high cost of labeling neurosurgical data has prohibited the development of these robust datasets. Our work sought to characterize available data sources used to develop AI models in neurosurgery and determine how to best develop future datasets. Our systematic review determined that the overwhelming majority of datasets used for AI in neurosurgery come from the spine domain, where trauma, tumor, and vascular datasets are <10% of data sources. Imaging (X-ray, CT, MRI) represents the most common data type available, most often labeled with diagnosis, segmentation, and detection/localization information. Very few datasets are labeled with outcomes, externally validated, or made available with either model weights or publicly available applications. The greater proportion of spine domain datasets may be driven by spine surgery being the largest subspecialty of neurosurgical case volume. However, there is a large clinical need for AI driven anatomical identification in tumor, vascular, functional, and pediatric subspecialties. For example, to enable large scale automation of cranial surgery, datasets of labeled cranial nerve and vascular structures will be essential. Similarly, while traditional imaging (e.g., X-ray, CT, MRI) represents the majority of data types, clinical data (e.g., surgical outcomes) and video data will be required across all fields to enable AI systems to place more active roles in surgical decision making and operation. Data privacy and informed consent for imaging and video datasets will become increasingly necessary for efforts building robust multi-institutional datasets.

The lack of outcome labels in addition to imaging data specifically limits the ability for models to be developed that can provide decision making support. Most datasets included imaging information. While these modalities are rich in spatial and anatomical information and well-suited for tasks such as segmentation, detection, and classification, they often lack accompanying clinical metadata and, more critically, patient outcome labels. This limitation significantly narrows the scope of AI model applications to static image interpretation tasks and precludes more clinically valuable use cases such as prognostic modeling [8], treatment response prediction [9], and decision support for personalized care [1]. Furthermore, there is a general lack of combining label types [7,10,11,12,13,14,15], such as diagnosis, classification, detection, and outcome. Without integration of outcome data into these data streams, it will be difficult to train models that can simulate the integrated clinical reasoning of clinicians and best make use of advances in foundational intelligence. Neurosurgeons are trained in decision making based on the longitudinal care of patients, and the ability to see the clinical outcomes of their surgical decision making. Without inclusion of outcome labels and surgical decision making parameters in dataset construction, AI model development for neurosurgery will be confined to task specific uses (e.g., anatomical identification). Incorporating clinically relevant data types (e.g., outcome, surgical parameters) into datasets may enable future work to develop surgical decision making support tools.

Our analysis revealed a pronounced domain imbalance across publicly available datasets. The spine domain accounted for over 80% of all included studies, while datasets related to cranial pathologies, including tumor, vascular, and trauma, were markedly underrepresented. This skewed distribution raises important concerns about the generalizability and scope of current AI efforts in neurosurgery. The dominance of spine relevant datasets is likely driven by the high volume of spine procedures, availability of standard imaging (i.e., X-ray, CT, MRI), and more standardized anatomical and pathological labeling. Neuro-oncology [16,17,18] and vascular domains [10,16,17] will likely involve multimodal data (e.g., radiology, pathology, genomics, operative video), which are not only harder to curate and annotate but also less frequently shared due to privacy, standardization, and institutional barriers. The absence of datasets from these areas limits the ability of researchers to build, test, and iterate on models tailored to critical, high-complexity neurosurgical decisions. To address this imbalance, future efforts should actively promote and fund intentional dataset construction in underrepresented domains. This includes incentivizing multi-institutional collaborations, developing annotation frameworks for complex data types, and standardizing protocols for sharing cranial neurosurgical data while preserving patient privacy. To enable such robust dataset creation, there is a need for cost-effectiveness analyses to be conducted to develop optimal strategies for labeling neurosurgical data while maximizing clinical effect.

Another major limitation preventing translation of AI to neurosurgery is the inconsistent use of performance metrics. Only 60% of segmentation baseline models were evaluated using the relevant performance metric of DICE score, whereas 48% of segmentation models used the inappropriate accuracy metric [18]. Even more important, it appears that the field is not correctly demanding the use of proper task relevant metrics as segmentation models that used accuracy as a metric received a comparable number of citations as those that used DICE (17.5% versus 20.2% of all segmentation citations) (Table 5). This issue is further supported by the lack of AUC and prevalence of accuracy in diagnostic prediction tasks. Guideline development in the field could encourage appropriate performance metrics which are essential for the safe translation of AI to neurosurgery.

A notable limitation in many dataset release papers is the incomplete reporting of foundational dataset metadata. Nearly one-fifth of included studies omitted the number of patients or institutional origin of data, reducing transparency and limiting reuse. Adoption of standardized reporting frameworks (e.g., Datasheets for Datasets) would improve reproducibility and comparability across neurosurgical AI datasets.

Our systematic review of neurosurgical data sources has several limitations that future work may address. First, we limited our search to PubMed and did not include work on IEEE Xplore or arXiv. While these sources may include datasets able to train ML models for neurosurgery, they are traditionally less accessible and used by the clinical audience. Furthermore, many arXiv papers have not been peer-reviewed. Nevertheless, future work should investigate the data availability, and robustness of the data for neurosurgical applications. A second limitation of the work is that we excluded studies with less than 100 data items. While this was important to exclude case reports and case series, given the rise of few-shot learning capabilities these smaller data releases may be useful for ML development and may be considered in future work. The current study is also limited in that it did not consider dataset collection methods, inclusion criteria, quality control measures, and reporting quality which are important aspects of both a dataset’s quality and applicability for future use that should be investigated in later studies. Analysis of baseline model performance is limited by heterogeneity of these same factors as well as inconsistent reporting of confidence intervals of metrics that limits interpretation of clinical significance of the prediction performance. Another limitation of the review is that some of these datasets may be composed of patient populations not generalizable to certain neurosurgical populations. We did not extract age, race, and sex of the cohorts comprising each study. Future studies can explore whether the distribution of these patient factors across datasets for ML in neurosurgery to better inform future dataset construction. This fairness issue has been studied in ML, and ideas of demographic parity, equal opportunity, equalized odds, statistical parity, and predictive parity should be applied to neurosurgical data sources. Similarly, investigation of the label quality of each source was not performed in the current study. Neurosurgical data is unique in that experts are required in a majority of cases for data labeling. Future studies should explore the accuracy of provided labels by label type to inform trust in both training and validating ML models using publicly available labeled data.

## 5. Conclusions

This systematic review highlights critical limitations in the current landscape of neurosurgical datasets used for AI model development. While most existing resources focus on spine imaging and address narrow tasks such as diagnosis and segmentation, they often lack outcome labels, external validation, and public availability, key factors needed to support clinical translation. Moreover, dataset imbalance, limited reproducibility, and the use of suboptimal performance metrics hinder the broader impact and generalizability of these models. To advance neurosurgical AI, future efforts should prioritize the intentional construction of high-quality, multi-institutional datasets that integrate imaging, clinical variables, and longitudinal outcomes. Such datasets should support external validation, enable reproducibility through code and model sharing, and target underrepresented domains such as neuro-oncology and vascular neurosurgery. This could potentially be catalyzed via guidelines. By addressing these gaps, the field can move toward developing robust, generalizable, and clinically actionable AI tools that meaningfully enhance neurosurgical practice.

## Figures and Tables

**Table 1:** Summary of published datasets.

Variable	Analysis
Neurosurgical subspeciality:	
Spine	72/86 (83.7%)
Tumor	3/86 (3.5%)
Vascular	4/86 (4.7%)
Trauma	6/86 (7.0%)
Other	1/86 (1.2%)
Data type(s) included:	
X-ray	32/86 (37.2%)
CT	18/86 (20.9%)
MRI	25/86 (29.1%)
PET	2/86 (2.3%)
Clinical	14/86 (16.3%)
Sensor	1/86 (1.2%)
Video	2/86 (2.3%)
Label type:	
Diagnosis	23/86 (26.7%)
Segmentation	31/86 (36.0%)
Detection/localization	18/86 (20.9%)
Intervention	5/86 (5.8%)
Grading	18/86 (20.9%)
Image	2/86 (2.3%)
Outcome	2/86 (2.3%)
Number of Patients	Mean: 756.4, SD: 925.8, Median: 100, Range: 1–4963
Multi-institutional data	23/86 (26.7%)
Number of institutions	Mean: 3.47, SD: 18.5, Median: 1, Range: 1–167
Includes baseline model	84/86 (97.7%)
Inference class of baseline model:	
Diagnosis	36/84 (42.9%)
Detection/localization	35/84 (41.7%)
Segmentation	29/84 (34.5%)
Intervention	4/84 (4.8%)
Measurement	7/84 (8.3%)
Grading	9/84 (10.7%)
Outcome	8/84 (9.5%)
Generative	1/84 (1.2%)
Model type:	
CNN	52/84 (61.9%)
Segmentation	26/84 (31.0%)
Linear	10/84 (11.9%)
Non-transformer NLP	3/84 (3.6%)
Transformer	3/84 (3.6%)
GAN	1/84 (1.2%)
Model externally validated	19/84 (22.6%)
Model/code publicly available	17/84 (20.2%)
Application publicly available	6/84 (7.1%)
Number of citations	Mean: 12.0, SD: 17.9, Median: 6.5, Range: 0–127

**Table 2:** Validation and availability of baseline model by model type.

Model Used	External Validation	Public Code	Public Application
Linear	0/10 (0%)	1/10 (10.0%)	1/10 (10.0%)
CNN	12/52 (23.1%)	11/52 (21.2%)	4/52 (7.7%)
Segmentation	6/26 (23.1%)	5/26 (19.2%)	2/26 (7.7%)
GAN	0/1 (0.0%)	0/1 (0.0%)	0/1 (0.0%)
Non-transformer NLP	1/3 (33.3%)	0/3 (0.0%)	1/3 (33.3%)
Transformer	1/3 (33.3%)	1/3 (33.3%)	0/3 (0.0%)

**Table 3:** Validation and availability of baseline model by primary data.

Model Used	External Validation	Public Code	Public Application
X-ray	10/31 (32.3%)	7/31 (22.6%)	3/31 (9.7%)
CT	5/17 (29.4%)	4/17 (23.5%)	3/17 (17.6%)
MRI	6/24 (25.0%)	6/24 (25.0%)	1/24 (4.2%)
PET	0/2 (0.0%)	1/2 (50.0%)	0/2 (0.0%)
Clinical	0/13 (0.0%)	2/13 (15.4%)	1/13 (7.7%)
Sensor	0/1 (0.0%)	0/1 (0.0%)	0/1 (0.0%)
Video	0/2 (0.0%)	0/2 (0.0%)	0/2 (0.0%)

**Table 4:** Frequency of performance metrics used to evaluate inference type.

Metric	Detection and Localization	Diagnosis	Generative	Grading	Intervention	Measurement	Outcome	Segmentation
Accuracy	12/26 (46.2%)	22/31 (71.0%)	0/1 (0.0%)	2/6 (33.3%)	3/4 (75.0%)	3/6 (50.0%)	5/5 (100.0%)	12/25 (48.0%)
AUC/AUROC	5/26 (19.2%)	9/31 (29.0%)	0/1 (0.0%)	2/6 (33.3%)	2/4 (50.0%)	0/6 (0.0%)	2/5 (40.0%)	2/25 (8.0%)
Dice	5/26 (19.2%)	3/31 (9.7%)	0/1 (0.0%)	0/6 (0.0%)	0/4 (0.0%)	2/6 (33.3%)	0/5 (0.0%)	15/25 (60.0%)
F1 Score	6/26 (23.1%)	10/31 (32.3%)	0/1 (0.0%)	2/6 (33.3%)	1/4 (25.0%)	0/6 (0.0%)	2/5 (40.0%)	3/25 (12.0%)
Gwet k	0/26 (0.0%)	1/31 (3.2%)	0/1 (0.0%)	1/6 (16.7%)	0/4 (0.0%)	0/6 (0.0%)	0/5 (0.0%)	0/25 (0.0%)
ICC	3/26 (11.5%)	1/31 (3.2%)	1/1 (100.0%)	0/6 (0.0%)	0/4 (0.0%)	2/6 (33.3%)	0/5 (0.0%)	1/25 (4.0%)
IOU	0/26 (0.0%)	0/31 (0.0%)	0/1 (0.0%)	0/6 (0.0%)	1/4 (25.0%)	0/6 (0.0%)	0/5 (0.0%)	3/25 (12.0%)
MAE	3/26 (11.5%)	0/31 (0.0%)	0/1 (0.0%)	0/6 (0.0%)	0/4 (0.0%)	2/6 (33.3%)	0/5 (0.0%)	0/25 (0.0%)
NPV	0/26 (0.0%)	4/31 (12.9%)	0/1 (0.0%)	0/6 (0.0%)	1/4 (25.0%)	0/6 (0.0%)	0/5 (0.0%)	1/25 (4.0%)
PR AUC	0/26 (0.0%)	0/31 (0.0%)	0/1 (0.0%)	0/6 (0.0%)	0/4 (0.0%)	0/6 (0.0%)	0/5 (0.0%)	1/25 (4.0%)
PPV	6/26 (23.1%)	15/31 (48.4%)	0/1 (0.0%)	2/6 (33.3%)	3/4 (75.0%)	1/6 (16.7%)	0/5 (0.0%)	5/25 (20.0%)
Sensitivity	16/26 (61.5%)	27/31 (87.1%)	0/1 (0.0%)	6/6 (100.0%)	3/4 (75.0%)	2/6 (33.3%)	4/5 (80.0%)	14/25 (56.0%)
Specificity	11/26 (42.3%)	18/31 (58.1%)	0/1 (0.0%)	5/6 (83.3%)	2/4 (50.0%)	2/6 (33.3%)	3/5 (60.0%)	10/25 (40.0%)

**Table 5:** Citations for inference type that use each performance metric.

Metric	Detection and Localization	Diagnosis	Generative	Grading	Intervention	Measurement	Outcome	Segmentation
Accuracy	136/732 (18.6%)	320/2109 (15.2%)	0/0	100/480 (20.8%)	42/214 (19.6%)	44/287 (15.3%)	19/58 (32.8%)	178/1020 (17.5%)
AUC/AUROC	45/732 (6.1%)	236/2109 (11.2%)	0/0	20/480 (4.2%)	46/214 (21.5%)	0/287 (0.0%)	7/58 (12.1%)	18/1020 (1.8%)
Dice	69/732 (9.4%)	33/2109 (1.6%)	0/0	0/480 (0.0%)	0/214 (0.0%)	69/287 (24.0%)	0/58 (0.0%)	206/1020 (20.2%)
F1 Score	48/732 (6.6%)	109/2109 (5.2%)	0/0	26/480 (5.4%)	13/214 (6.1%)	0/287 (0.0%)	13/58 (22.4%)	37/1020 (3.6%)
Gwet k	0/732 (0.0%)	26/2109 (1.2%)	0/0	26/480 (5.4%)	0/214 (0.0%)	0/287 (0.0%)	0/58 (0.0%)	0/1020 (0.0%)
ICC	13/732 (1.8%)	0/2109 (0.0%)	0/0	0/480 (0.0%)	0/214 (0.0%)	1/287 (0.3%)	0/58 (0.0%)	0/1020 (0.0%)
IOU	0/732 (0.0%)	0/2109 (0.0%)	0/0	0/480 (0.0%)	0/214 (0.0%)	0/287 (0.0%)	0/58 (0.0%)	22/1020 (2.2%)
MAE	13/732 (1.8%)	0/2109 (0.0%)	0/0	0/480 (0.0%)	0/214 (0.0%)	1/287 (0.3%)	0/58 (0.0%)	0/1020 (0.0%)
NPV	0/732 (0.0%)	151/2109 (7.2%)	0/0	0/480 (0.0%)	0/214 (0.0%)	0/287 (0.0%)	0/58 (0.0%)	0/1020 (0.0%)
PR AUC	0/732 (0.0%)	0/2109 (0.0%)	0/0	0/480 (0.0%)	0/214 (0.0%)	0/287 (0.0%)	0/58 (0.0%)	43/1020 (4.2%)
PPV	21/732 (2.9%)	275/2109 (13.0%)	0/0	26/480 (5.4%)	42/214 (19.6%)	0/287 (0.0%)	0/58 (0.0%)	26/1020 (2.5%)
Sensitivity	204/732 (27.9%)	532/2109 (25.2%)	0/0	141/480 (29.4%)	42/214 (19.6%)	86/287 (30.0%)	13/58 (22.4%)	260/1020 (25.5%)
Specificity	183/732 (25.0%)	427/2109 (20.2%)	0/0	141/480 (29.4%)	29/214 (13.6%)	86/287 (30.0%)	6/58 (10.3%)	230/1020 (22.5%)

## Data Availability

All extracted data items are included in Supplemental Table S1. Any other data is available upon request to the corresponding author: ethan.schonfeld@stanford.edu.

## Supplementary Materials

The following supporting information can be downloaded at: https://www.mdpi.com/article/10.3390/jcm14165674/s1, Table S1: Summary of Included Studies and Associated Data Items. Table S2: Analysis of Bias in Included Studies.
